# Climate change and healthy ageing: An assessment of the impact of climate hazards on older people

**DOI:** 10.7189/jogh.14.04101

**Published:** 2024-05-24

**Authors:** Matthew Prina, Nusrat Khan, Samia Akhter Khan, Jorge Castro Caicedo, Anna Peycheva, Veri Seo, Siqi Xue, Ritu Sadana

**Affiliations:** 1 Population Health Sciences Institute, Newcastle University, Newcastle upon Tyne, England, UK; 2 Department of Global Health & Social Medicine, King’s College London, London, England, UK; 3 Department of Health Service & Population Health, King’s College London, London, England, UK; 4 East London NHS Foundation Trust, London, England, UK; 5 Department of Child and Adolescent Psychiatry, King’s College London, London, England, UK; 6 Department of Psychiatry, Cambridge Health Alliance, Cambridge, Massachusetts, USA; 7 Department of Psychiatry, University of Toronto, Toronto, Ontario, Canada; 8 World Health Organization, Geneva, Switzerland

## Abstract

**Background:**

Climate change not only directly impacts older people’s longevity but also healthy ageing, which is the process of maintaining physical and mental capacities while optimising functional abilities. The urgency to address both population ageing and climate change necessitates a rethink and assessment of the impact of climate change on older people. This includes identifying what can be done to anticipate, mitigate and adapt to climate change and engage older persons.

**Methods:**

A review of climate change and healthy ageing forms the basis of evidence in this report. We developed a comprehensive search to assess current literature, combining terms related to ageing and climate change across four major data sets and assessing articles published up to the end of 2021.

**Results:**

We summarised the current and future impact of climate change on older people and developed a framework identifying climate change impacts on older persons, recognising social and environmental determinants of healthy ageing. Major hazards and some key exposure pathways include extreme temperatures, wildfire, drought, flooding, storm and sea level rise, air quality, climate-sensitive infectious diseases, food and water insecurities, health and social care system displacement, migration, and relocation. Strategies to address climate change require interventions to improve systems and infrastructure to reduce vulnerability and increase resilience. As a heterogeneous group, older people’s perceptions of climate change should be integrated into climate activism. Increasing climate change literacy among older people and enabling them to promote intergenerational dialogue will drive the development and implementation of equitable solutions. Pathways may operate via direct or indirect exposures, requiring longitudinal studies that enable assessment of exposures and outcomes at multiple time points, and analyses of cumulative impacts of hazards across the life course.

**Conclusions:**

The lack of systematic reviews and primary research on the impact of most climate hazards, except for heat, on older people is apparent. Future research should include outcomes beyond mortality and morbidity and assess how older people interact with their environment by focusing on their capacities and optimising abilities for being and doing what they value.

Extreme heat, natural disasters (including floods, typhoons, and hurricanes) and changing patterns of infection due to climate change are predicted to lead to an additional 250 000 deaths a year worldwide from 2030 to 2050 [1]. Defined as the ‘greatest threat to societies and global health of the 21st century’, climate change is characterised by a long-term shift in weather patterns at a global level [2]. Climate change is the result of human activities, such as the burning of coal, gas, and oil, which produce carbon and nitrous oxide that linger in the atmosphere, or changes in land use as well as agricultural practices, such as deforestation, and increased livestock farming [1]. Those drivers are not only modifying the environments in which we live but also have direct repercussions on our lives and health.

Climate change is an existential threat to humans, necessitating urgent and comprehensive action [3]. The combined global challenges of climate change and population ageing exemplify the paramount issues of this century, requiring simultaneous consideration and action, with both having significant impacts on health [4,5]. Climate change impacts every life, but older people are disproportionally affected by these changes [6]. This is due to a heightened vulnerability of older individuals, which stems from physiological, social, and contextual factors raising their risk of adverse health effects due to climate change [7] (**Figure 1**). For example, during Hurricane Katrina, 71% of the fatalities were older people, despite them only being 15% of the population [8]. A recent study assessing floods across 761 communities worldwide also found stronger associations between floods and mortality risk in sites with larger proportions of older people, combined to those that had a smaller older population [9]. Higher mortality risk among older people, compared to younger, have also been identified in the case of heat [10], with a 45-year study in Scotland reporting the oldest age group (85+) as the most affected by extreme cold and heat exposure, particularly for cardiovascular mortality [11]. As reported later in this paper, increased mortality is not the only outcome that highlights the increased vulnerability of older people. Despite this, as a group, older persons have often been neglected in studies on the impact of climate change. This is a major oversight for older persons living in all regions and countries, not only in high-income countries: already two-thirds of older people live in middle-income countries. Moreover, the proportion of older persons globally is increasing; by 2030, one in six people will be 60 years of age or older globally, and by 2050, one in five will represent over two billion older people [1,12].

**Figure 1:**
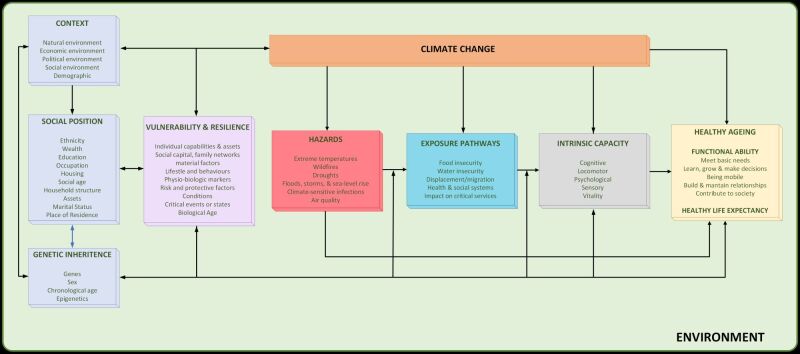
Framework of the impact of climate change on older people.

Global policies are taking note and can be used as a platform to address the needs and rights of all older people. In December 2020, the United Nations (UN) launched the Decade of Healthy Ageing for 2021–2030, uniting various stakeholders to enhance the well-being of older people in line with the Sustainable Development Goals (SDG) – with healthy ageing defined as ‘the process of developing and maintain the functional ability that enables well-being in older age’ [12]. This is an opportunity to rethink and assess the impact of climate change on older people, as all UN Member States have endorsed that the environment plays a crucial role in promoting healthy ageing [13,14]. Climate change impacts older individuals and the process to optimise healthy ageing affects not only longevity but people’s abilities to be and do what they value [10,11]. This includes people’s ‘intrinsic capacities’ and a person’s interaction with their local environment including the natural environment. The interaction between the person and their environment enables or hinders people to function in families, communities or society with supportive environments optimising a person’s ‘functional ability’ at any level of ‘intrinsic capacity [7,12]. Together, these shape how individual older people experience well-being.

The overall aim of this paper is to assess the state of the evidence on the impact of climate change on older people. By constructing this conceptual framework, we aim to create a foundation for discerning adaptation strategies, policies, and actions that can mitigate the present and future consequences of climate change on older people and healthy ageing. Specifically, we aim to: 1) assess the impact of different climate hazards on older people and identify the exposure pathways; 2) develop a conceptual framework to summarise the impact of climate change on older people; 3) identify vulnerabilities at an individual and societal level that put older people at a higher risk of being impacted by climate change.

## METHODS

The evidence discussed in this report was identified by using a combination of search strategies (**Box 1****)**, which helped us in developing a theoretical framework.

Box 1 **Methodology of screening and identifying relevant articles**

The searches were carried out up to December 2021 and by screening the first 50 records on Google Scholar. After deduplication, 2577 articles underwent title and abstract screening (this includes the articles from Google and Google Scholar) for the primary paper screening. For the screening of reviews, a total of 3242 articles were initially screened after deduplication. Reference lists of these papers were also reviewed, and relevant articles were selected. Given the lack of evidence specific to older people, we did not limit those searches to older people.

**Step 1: Rapid review**

*Data sets:* Medline, EMBASE, PSYCINFO, GLOBAL HEALTH (up to 2021), Google Scholar (first 50 records), Google (first 50 records)**

Search terms:Climate change and (older OR older adults OR agein* OR agin* OR aged)

**Step 2: Reviews of reviews**

Data sets: Medline (filter: 2011-2022) and Google Scholar (first 50 records)* ^&^ **

Search terms:

1- climate AND review AND wildfire* (n = 100 results in medline)

2- climate AND review AND drought* (n = 810 results in medline)

3- (climate change* OR global warming) AND (infectious disease* OR communicable OR tropical disease* OR zoonotic OR vector?born* OR water?born* OR food?born*) AND (review) (n = 950 in medline)

4- (climate change* OR global warming) AND (review) AND (flood* OR sea?level OR typhoon* OR hurricane*) (n = 519 in medline)

5- (Heat OR heat wave* OR warm*) AND (health OR health risk*) AND (climate chang* OR global warming) AND (review) (n = 763 in medline)

**Step 3: Screening of other grey literature sources.**

On top of searching Google, we also screened for potentially relevant reports from key organisations including IPCC, WHO, United Nations, Help Age International, etc.

*Separate searches conducted for heat, droughts, wildfires, flooding, sea-level rises, and climate-sensitive infectious diseases

** Not limited to only older people as there would be few reviews that focused specifically on the impact of hazards in later life. However, we were primarily only interested in papers that focused on older people or where the evidence was transferrable to later life, defined as:

1. Studies/articles referring to older people using a pre-defined cut-off age.

2. Studies that reported a mean age of 60 years or above

3. Studies reporting specific estimates stratified by age groups, including an older age group.

## RESULTS

Below, we introduce the main hazards and some key exposure pathways identified from the search.

### Impact of climate change on older people: Conceptual framework

Climate change impacts older people’s capacities and abilities in diverse ways through a complex network of interconnected pathways. We have conceptualised a framework – a theory of change – on the impact of climate change on older people (**Figure 1**). This is a further development of the initial framework for healthy ageing that was created by Sadana et al. [15] integrating a social and environmental determinants of health model and the International Classification of Functional, Disability and Health (ICF). This approach emphasises the broader environmental context, encompassing climate dynamics, and involving systematic organisation and evaluation to promote healthy ageing. Rooted in the biopsychosocial model, the ICF facilitates the precise assessment of interventions at individual and societal levels, considering the influence of climate and environmental determinants on health outcomes [16]. Terms that are used throughout are based on the definitions of the Intergovernmental Panel on Climate Change (IPCC) [17], the World Health Organization (WHO) Baseline Report for the Decade of Healthy Ageing [18] and WHO’s strategy on health, environment and climate change [19] (**Box 2**).

Box 2 **Key terminologies**

**Healthy ageing** is defined as ‘the process of developing and maintaining the functional ability that enables well-being in older age.’ There are three main components of healthy ageing: functional ability, intrinsic capacity, and environment [18].

**Intrinsic capacity** represents every mental and physical capacity that a person can resort to. It includes several domains, including cognition, locomotor capacity (physical movement), psychological capacity, sensory capacity (hearing and vision), and vitality (energy and equilibrium) [18].

**Environments** are where people live and conduct their lives. They include natural and built environments, but also communities and broader societies and all factors within them [18].

**Functional ability** is the combination of the intrinsic capacity of a person, the environment they live and their interaction with the environment. It ‘enables people to be and to do what they have a reason to value’ [18]. It includes several domains: a) meeting basic needs to ensure an adequate standard of living; b) learning, growing, and making decisions; c) being mobile; d) building and maintaining relationships; and e) contributing to societies.

**Healthy life expectancy** is an indicator reflecting the years spent in good health, which combines morbidity (disease, illness or injury) and mortality (death) [20]. It is an important outcome indicator that has been endorsed in the Decade of Healthy Ageing, and is also WHO’s overall indicator to measure impact of its work on population health [18].

**Hazard** has been defined as ‘the potential occurrence of a natural or human-induced physical event or trend that may cause loss of life, injury or other health impacts, as well as damage and loss to property, infrastructure, livelihoods, service provisions, ecosystems and environmental resources’ [20].

**Exposure** is defined as ‘the presence of people, livelihoods, species or ecosystems, environmental functions, services and resources, infrastructure, or economic, social or cultural assets in places that could be adversely affected.’ In this section we mainly talk about exposure pathways to highlight factors that coexist with each other and with hazards and that can positively or negatively influence outcomes [20].

**Impact** has been defined as ‘adverse outcomes on natural and human systems, where risks result from the interactions of climate-related hazards, exposure, and vulnerability.’ Impacts can affect humans, natural, social and built environments [21].

**Vulnerability** is defined as ‘the propensity or predisposition to be adversely affected. Vulnerability encompasses a variety of concepts and elements including sensitivity or susceptibility to harm and lack of capacity to cope and adapt’ [20].

Certain hazards can compound each other, while others result from preceding hazards, for instance, droughts arising from extreme temperature and rainfall patterns. For simplicity, we have categorised all hazards together and have concentrated solely on primary hazards with the potential to impact older individuals, acknowledging the existence of other hazards. The ramifications of climate change on healthy ageing can manifest through intricate, either direct or indirect, exposure pathways. These hazards exert their influence on older individuals by directly shaping their healthy life expectancy and by affecting fundamental aspects of healthy ageing, including intrinsic capacity, functional ability, and the environment.

Certain hazards can compound each other, while others result from preceding hazards, for instance, droughts arising from extreme temperature and rainfall patterns. For simplicity, we have categorised all hazards together and have concentrated solely on primary hazards with the potential to impact older individuals, acknowledging the existence of other hazards. The ramifications of climate change on healthy ageing can manifest through intricate, either direct or indirect, exposure pathways. These hazards exert their influence on older individuals by directly shaping their healthy life expectancy and by affecting fundamental aspects of healthy ageing, including intrinsic capacity, functional ability, and the environment.

It is also imperative to underscore that the older population is heterogeneous, displaying substantial diversity within it. Consequently, not all older adults are equally vulnerable and characteristics (e.g. place of residence, social position, national and political environment, etc.) across subgroups should be considered when developing policies.

### Major hazards

#### Extreme temperatures

Globally, ambient temperatures have risen by more than 1°C compared to pre-industrial levels, a change primarily driven by human activities, deteriorating air quality, and the accumulation of greenhouse gases that enhance the greenhouse effect [19,22]. These temperature shifts have profound implications for human health, especially among older individuals (Figure S1 in the **Online Supplementary Document**). Thermoregulatory abilities diminish with age, making older adults more susceptible to extreme temperatures [23]. Additionally, the ability to perceive temperature changes decreases with age, compounding the risk of hypo- or hyperthermia with continued exposure to extreme temperatures [24]. Consequently, there has been a rise in morbidity and mortality linked to extreme heat, a trend expected to continue in the coming years. High temperatures can lead to increased overall morbidity and mortality in several ways. For instance, heart failure can result from heatstroke [25,26]. Dehydration, often exacerbated by inadequate access to clean water, has been linked to increased incidence of acute kidney injury and renal failure. Heatwaves also worsen respiratory health, as evidenced by reports of increased respiratory-related hospital admissions during heatwaves in Europe and North America [27]. Many older people have pre-existing medical conditions such as cardiovascular disease, diabetes, and asthma, which put them at high risk for heat-related morbidity and mortality. Moreover, heat waves limit mobility and outdoor activities for older individuals, exacerbating sedentary lifestyles and chronic conditions [28]. Furthermore, coping with extreme heat often necessitates adjusting indoor temperatures, including the use of air conditioning if available. This can result in increased electricity bills and contribute to higher CO_2_ emissions, accelerating climate change. For many older individuals without family support, these expenses can be financially burdensome, heightening their vulnerability to the adverse effects of extreme temperatures.

### Wildfires

Wildfires pose a high threat to humans and are increasing in frequency (Figure S2 in the **Online Supplementary Document**) [29]. Wildfires disproportionately impact older people in several ways. Wildfires can affect the environments where older people reside, bringing several respiratory complications for older people. A meta-analysis using data from 19 European and North American countries by Huang and colleagues reported that several particulate matter (PM), some of which are associated with wildfires, were significantly related to lung cancer [30]. Children, older people, and those with underlying conditions seem to be at a higher risk compared to other adult populations. A study from Brazil reported that short-term exposure to wildfire related PM2.5 is associated with increased risks of all-cause, respiratory, and cardiovascular hospital admissions. Children (zero to four years), and older people (≥80 years) had the highest relative risks of hospitalisation per 10 µg per cubic meter (μg/m^3^) increase in wildfire-related PM2.5, 4.88% (95% confidence interval (CI) = 4.47–5.28), and 3.70% (95% CI = 3.20–4.20) respectively, with adults aged between 30 and 39 only having 0.83% (95% CI = 0.44–1.23) [31]. It has been suggested that this could be the result of weaker immune systems and pre-existing health conditions, such as asthma, which could increase the susceptibility to the respiratory impact of wildfires [32]. Due to air pollution, wildfires have effects on older people’s cardiovascular health. Evidence from Australia shows that cardiovascular mortality rates increased during days when wildfires appeared [29]. Older people are also more likely to use emergency services due to cardiac arrest and become hospitalised due to ischaemic heart disease shortly after wildfires, compared to younger people [33]. Wildfires can also have a negative psychological impact on older people. Wildfires usually destroy houses, lifestyles, the former living environment and communities of older people, preventing ageing in place [34]. Older people may require more time or special support in case of evacuations, and power shortages and increased risk of electrocution from fallen lines may also affect health care facilities and older people’s households located in rural areas [29].

### Drought

Droughts have been defined as extreme climate events in which the reduction of water, precipitation and an increase in air temperature are observed [35]. The increased duration of droughts will have various negative implications for the global population, especially for older people (Figure S3 in the **Online Supplementary Document**). Droughts particularly affect the environment of older people living in rural areas. In northern Ghana, for example, droughts were reported to reduce the fertility of the soil, reduce harvest quantities, and destroy property and livestock [36]. These effects of droughts can devastate older farmers from low- and middle-income countries (LMICs) due to their economic effects and can also increase the risks of malnutrition [37]. Charnley and colleagues also found that droughts in the African continent may create ideal environments for the proliferation of Vibrio cholerae, the bacteria responsible for cholera disease, which can be highly detrimental to the health of older people, who have a higher risk of developing water-borne diseases [38].

### Flooding, storms, and sea level rise

Hydro-meteorological disasters affect the ageing population in a multitude of ways (Figure S4 in the **Online Supplementary Document**). A recent assessment of the needs of older people after the 2022 Pakistan floods highlighted a stark picture, with many older people left in need of cash, food, housing, medicines, and hygiene items [39]. Similarly, a case study from the USA reported a disproportional increase in emergency department visits and hospitalisations among older people in the week following Hurricane Sandy [40]. Hydro-meteorological disasters contribute to morbidity and mortality in older people through both direct and indirect means. Directly, reasons for death may include drowning, serious blunt trauma, and electrocution [41]. In addition to being vulnerable to direct impacts from water and flooding, older people are further at risk for other indirect health consequences. Floods have been linked to increased rates of fatal and non-fatal acute cardiovascular events, including myocardial infarction, Takotsubo syndrome (acute stress-induced cardiomyopathy), and cerebrovascular accidents, to which older people are particularly susceptible [41,42]. Hydro-meteorological disasters can also lead to an increased risk of numerous infectious diseases [43], and disasters can also impact mental health among the older population [44].

### Air quality

Air pollutants, such as ozone, black carbon, and sulphates, can lead to a change in climate. At the same time, increasingly warmer days, precipitation, and wind pattern changes can lead to the entrapment of air pollutants, such as ozone and particulate matter (e.g. soot, dust, dirt) in the lower atmosphere [45]. Older people are highly susceptible to the effects of poor-quality air (Figure S5 in the **Online Supplementary Document**). Studies have shown that small PM clearance reduces with increasing age, which is particularly problematic given that PM has been associated with both acute and chronic health effects, including asthma incidence and aggravation, chronic obstructive pulmonary disease (COPD), cancer, cardiovascular disease, and pneumonia [46]. A meta-analysis of the health risks associated with short-term exposure to PM showed that older people have a higher risk of death (0.64%) compared to younger populations per 10 μg/m^3^ increase of PM. This included data from different European countries, North America (Canada, Mexico, and the USA), South America (Brazil and Chile) and Asia (China, India, and South Korea) [47].

### Climate-sensitive infectious diseases

While the global burden of disease has shifted from communicable to non-communicable causes in the last century, infectious diseases remain an important source of morbidity and mortality in older people, accounting for 5–6% of disability-adjusted life years (DALY) in 2019 and underlined by the COVID-19 pandemic [48]. Older people are both more prone to certain infections and likely to be diagnosed late due to age-related immunosuppression, being malnourished, or having other health conditions [37,49]. The WHO estimates that climate change will be responsible for over 60 000 additional deaths from malaria and dengue in 2030, as well as 48 000 additional deaths from diarrhoeal illnesses globally [14].

Climate-sensitive infectious diseases (CSIDs) are infections that are vulnerable to changing climatic factors such as increased temperatures and precipitation (Figure S6 in the **Online Supplementary**). One meta-analysis found that a 1°C increase in global temperatures was associated with overall infectious morbidity among older people, but more evidence is needed to establish the magnitude of the effect [25]. Vector and waterborne diseases are the most likely to be impacted by climate change [50]. Malaria, for example, is a major vector-borne disease. Malaria in older people is associated with a higher risk of neurological damage and death in older adults. Dengue, another vector-borne disease on the rise with climate change, is associated with haemorrhagic fever and end-organ damage. Older people could, therefore, be at proportionately greater risk as climate change increases their overall incidence [51,52]. Waterborne gastrointestinal infections are a particularly important cause of illness in older people due to age-related immunosuppression, decreased thermoregulatory ability, and sensitivity to dehydration [53]. In terms of foodborne CSIDs, *salmonella* and *campylobacter* have peak incidences in older people, who are also more likely to be hospitalised and die after illness compared to younger populations [54]. Norovirus is a key illness for older people who are more likely to have prolonged hospitalisation and death caused by norovirus, with a fatality rate higher than >200% that of young children [55].

### Food and water insecurities

Food insecurities are driven by a multitude of factors: warmer weather can lead to poorer crops and greater food spoilage. This is problematic as many older people living in LMICs continue working until later in life and often work as farmers or rely on subsistence agriculture [56]. It has been estimated that up to 28% of smallholder farmers globally are aged over 55. Many older people live on fixed incomes and will not be able to afford rapid price increases. It has also been reported that when food is scarce, households are more likely to allocate limited food supplies to younger members of the family compared to older members [57,58]. All these issues could lead to malnutrition disproportionately affecting older women. High temperatures are also associated with reduced food safety and can increase pathogens in food and seafood, once again disproportionally affecting older people due to pre-existing co-morbidities and reduced immune systems [59]. A recent review of the literature has linked water insecurity with diarrhoeal incidence, chronic kidney disease, increased blood pressure, reduced cognitive performance and dehydration [60]. Older people are increasingly vulnerable to these changes, as they are already underserved by existing facilities and services [61]. Dehydration in later life is burdensome, and several studies have shown that there is a strong association between being dehydrated and having negative health outcomes, including mortality, frailty, cardiovascular disease, cognitive problems, oral health and poorer hospital outcomes [60].

### Health and social care systems

Climate change is having and will have a large impact on health and social care systems. Many of the hazards described above will result in acutely increased hospitalisations after extreme events and the use of already stretched and overburdened health systems. Low- and middle-income countries will be disproportionally affected by this increase in burden due to poverty, malnutrition, lack of resources to address health needs, and poor underlying infrastructure. Displacement from home will also disrupt access to health and social care systems, with a report from the UK highlighting a substantial impact of evacuation efforts on well-being and health [62]. Social care for older people often relies on both formal and informal caregiving. Both are being affected by climate change. Urban migration of younger generations in areas impacted by climate change could result in reduced support ratios and informal caregiving for those older people who are left behind and in need of informal care support [63]. This lack of intergenerational support structures may lead to increasing loneliness among older people, further exacerbating the threats to healthy ageing [64].

### Displacement, migration, and relocation

Climate change-related displacement and migration refers to the movement of individuals and communities due to changing climate conditions that threaten life, livelihood, or well-being [61,65,66]. Migration is often a last resort adaptation to climate change. However, a person’s ability to migrate is limited by mobility and resources. Older people, who are more likely to have socioeconomic vulnerabilities, locomotor limitations, underlying health conditions, and greater social care needs, are often the least able to relocate in approaching climate threats [65,67]. Case studies in Bangladesh, Nepal, and Pakistan have shown that older people are among the vulnerable subgroups often left behind in climate migration [68]. In cases of successful relocation, older people may be more vulnerable to other exposure pathways and risks of climate change, such as food insecurity or loss of access to critical services. Migrant groups generally have worse health outcomes due to dangers in transit and disrupted health and social care [69,70]. Evacuations pose special risks to older people who are frail, medically complex, or live in group care settings, as needed medications, medical equipment, and medical records may not be accessible [63].

### Vulnerabilities and resilience

While climate change affects all older individuals to some extent, it has a more pronounced impact on specific subpopulations of older people, attributable to their characteristics and broader determinants of health. When these factors are anticipated to result in deleterious consequences, they are designated as ‘vulnerabilities’ and, when positive or building up reserve, as ‘resilience’. These vulnerabilities often overlap and coexist, working together dynamically and synergistically to perpetuate unequal health outcomes related to climate change.

Older people comprise a diverse population with varying experiences of risk factors on individual, social, and contextual levels. Vulnerabilities among older people can be seen as the result of how risk factors, sensitivity, and coping capacity interact when confronting climate threats [71]. There is evidence highlighting that factors affecting healthy ageing, such as social determinants of health, are evolving and older persons’ vulnerabilities have increased due to climate change impacts [19]. For example, several studies have identified a key role in socio-economic markers of position, such as education and wealth, as key drivers of inequalities in healthy ageing [72,73]. At the same time, climate change is amplifying existing inequalities by exacerbating vulnerabilities in people with lower incomes. This group of people often have lower awareness of climate change, with more limited resources to access knowledge on and implement adaptation strategies. Older people with low socio-economic positioning often live in poorer housing conditions and have less access to health services which include preventive, promotion, treatment, rehabilitation, and palliative care [74,75]. In the event of a climate crisis, this can lead to greater relative losses compared to younger or more affluent segments of the population. Thus, a vicious cycle occurs, whereby poverty increases older adults’ vulnerability to climate hazards, which in turn exacerbates economic inequalities [76,77]. In this paper, one objective is to explore specific vulnerabilities and offer relevant examples when possible. In essence, the climate crisis exacerbates pre-existing health disparities, as identified in some examples in **Table 1**.

**Table 1:** Vulnerabilities of older people and interactions

Major factors	Interaction factors	Examples
Sociodemographic characteristics	Gender	Women are at increased vulnerability to climate events with age if they outlive their male peers [77–79]
	Education	Lower education status is closely linked to lower socio-economic status, which is further related to poorer health outcomes in older people [80,81]
	Occupation	Occupational heat stress in construction work, mining, factory work, etc., can pose a serious health threat, leading to higher mortality and morbidity [82,83]
	Ethnicity	Barriers to access health care, health inequalities and discrimination for minority ethnic groups can further exacerbate vulnerabilities to the effects of climate change, particularly in older people from ethnic minorities [84,85]
	Poverty	Poverty limits people’s access to adequate health care services and access to knowledge, which can be detrimental to older adults’ functional ability and healthy longevity [5,86]
Health factors	COVID-19	In England, an excess of ( ~ 88%) of total cumulative all cause excess mortality from the COVID-19 pandemic occurred among individuals the 65+ age group [87,88]
	General health and comorbidities	With climate change, older people with pre-existing health conditions and with lower intrinsic capacity levels are at much higher risk of negative consequences than the general population [87,89]
	Cognition and mental health	During heatwaves, people living with dementia are at an increased risk of hospitalisation, and those with pre-existing mental health conditions, such as psychoses and substance misuse, may also be at an increased risk of heat-related mortality [90,91]
Social and contextual factors	Social links/ connectedness	A review from Nepal has demonstrated that the migration of active and educated individuals to urban areas has left older people with fewer adaptive resources and social support, increasing their susceptibility to the adverse impact of climate change [81,92]
	Built environment and housing	In Portugal, larger wildfire-burned areas tend to be more inland and associated with where ageing communities live and poorer housing infrastructure [93,94]
	Geographical location	Many climate-sensitive infectious diseases are likely to spread in Central and South America, South Asia, and Africa, imposing a burden on the already fragmented health care infrastructure in low HDI regions [95]
	Political situation, economic factors, armed conflicts	Political and demographic changes alongside socio-political instability, which lead to migration and displacement, disproportionately impact the lives of older people [63,96]

COVID-19 – coronavirus disease 2019, HDI – human development index

## DISCUSSION

### Implication for mitigation and adaptation

Despite increasing global awareness of the interplay between climate change and public health, current efforts fall short of achieving a sustainable recovery and safeguarding people from the mounting health risks associated with climate change [97]. As the majority of mitigation approaches focus on reducing the drivers of climate change (i.e. greenhouse gases) and are therefore not specific to older people, in this section, we will primarily discuss potential adaptation strategies.

WHO conducted a consultation with older adults to identify research questions addressing healthy ageing involving more than 1500 people in 77 countries [1] and documented many related to climate, extreme environmental events, pollution hazards, and the impact on housing, indoor and outdoor infrastructure, as well as the increased need for information and new technologies [98]. Several documents on this topic, including some guidelines from different organisations, already exist. Although these are not specific recommendations for older people, their integration could still be important to lessen the impact of climate change on this group. These include the WHO Global Strategy on Health, Environment, and Climate Change [19], WHO’s Housing and Health Guidelines [99], the Compendium on Health and Environment [100], the Lancet’s Countdown on Health and Climate Change series [101], the Sustainable Development Goals [102], the UNFCC reports [103] and the Paris Agreement itself [103].

Mitigating vulnerabilities, especially those to which older individuals are more predisposed, and enhancing resilience, constitute pivotal strategies to bolster preparedness and curtail the adverse effects of climate change on healthy ageing. Some important strategies suggested by different international bodies and scientists across the globe are listed in **Table 2**.

**Table 2:** Strategies for mitigation and adaptation

Key areas	Recommended strategies
Building intrinsic capacity and functional ability	Intrinsic capacity and functional ability can be built and optimised throughout the life course, with policy actions developed to shape those determinants of healthy ageing [102]
	Developing interventions following the WHO Integrated Care for Older People (ICOPE) guidelines adapted to contexts which are more prone to climate change [103,104]
	Increase accessibility to health services to older populations and increase the budget allocated to tackle health deteriorations in climate events [105]
Improving systems, communities, and infrastructures	
*Housing*	To protect older residents, houses and living facilities should ensure adequate ventilation and insulation [106]
	In heat-prone areas, buildings should utilise reflective surfaces and avoid heat-trapping materials like concrete to minimise heat absorption [107]
	Age-friendly housing codes with wider doorways and large staircases or stairlifts to support everyday mobility and facilitate quick evacuation during disasters [63]
*Cities and surrounding infrastructure*	Increased greenspaces to protect against extreme temperatures, mitigate pollution, provide shade, and offer aesthetic spaces for multiple types of activities for older people [108,109]
	Ensure walking paths are smooth surfaced, and well-lit to mitigate falls and safety concerns [109]
	In areas prone to heatwaves, heat shelters around the community should offer both physical safety and areas for social connection [56]
*Transport and mobility*	Ensure strong transport networks for timely evacuation and aid delivery, particularly for older people [56]
	In flood-prone areas, roads could be fitted on either side with bioswales, which are mulched and vegetated channels that facilitate water runoffs [110]
*Energy and resource poverty*	Local governments/municipalities to introduce new technologies like solar panels to provide low-emission, clean-energy power to homes [111]
	Provide information, technological training, or finances to older people to access renewable energy, which may help mitigate climate change and secure energy sources [56]
*Agriculture, food, and water security*	Utilise and support older people’s knowledge on how to deal with agricultural losses during extreme climatic events, such as planting different crops and changing farming techniques [56]
	Provide information and economic aid to more vulnerable older people to ensure food security [112]
	Provide information and training on recycling water, practicing adequate water storage techniques, and restricting excessive water use within the community [113]
*Health and social systems*	Adaption and contextualising of WHO ‘Operational framework for building climate resilient health systems’ for older people [114]
	Develop institutional policies and enhance the knowledge of health practitioners to facilitate the discussion of climate change with patients [115]
	Introduction of new technology, telehealth, remote monitoring, precision health, and artificial intelligence [116]
*Pathogen control*	Execute wider municipal programmes to control pathogen proliferation and improve sanitation to mediate the risk of CSID in older people [56]
	Public health programmes should be implemented in areas vulnerable to climate-sensitive vectors, educate older people and providers, and encourage behavioural changes
	Maintaining the quality and supply of community water sources is important for older people who are vulnerable to gastrointestinal illnesses.
*Early warning systems and disaster mitigation*	Early warning systems should be able to identify and send targeted messages to older people, particularly those with other vulnerabilities, including older persons with declines in hearing and vision
	Ensure availability and accessibility of communication technologies especially targeted to socially isolated older people [107,111]
	Early warning systems should use diverse and accessible communication avenues (e.g. public radio) to maximise reach to older people

### Potential initial action

To create effective and pertinent strategies for climate change prevention and mitigation, all affected communities need to participate, regardless of age, as emphasised by the UN Human Rights Council in 2021 [63]. Understanding the perception of climate change among older individuals is a crucial area to investigate for effective action. In 2010, the Stockholm Environment Institute reported that the baby boomer generation, currently aged 58–76, had the highest carbon footprint in the UK standing at 13.5 CO_2_ tonnes per capita [117], with this footprint driven to meet basic needs rather than having luxury lifestyles [118]. It has been argued that older people in the USA have various objections to climate change action, such as the idea that it is too late to reverse climate change [119], although other studies found no major differences in perceptions between younger and older cohorts [120]. One of the first initial actions is to change the perception of older individuals so that they recognise their role as significant drivers and contributors to climate change action.

A recent survey involving 1.4 million participants from 50 countries underscored that the individual's level of education was the most significant influencer of public opinion regarding climate change [121]. To comprehend why older generations are not as actively involved in climate action as younger ones, it is imperative to consider additional barriers (including individual, structural, and societal barriers) beyond lower education levels and a lack of personal exposure to the adverse effects of climate change, which may limit the scope for older individuals' engagement in environmental initiatives.

Engaging in climate action not only fosters healthy ageing but also positively impacts community well-being and environmental preservation. Older people are inherently motivated to contribute to society. The inclination of older populations to assist during crises with substantial environmental consequences or other emergencies, even at the risk of their well-being, is a remarkable characteristic. Scholars have proposed that fostering a sense of generativity and emphasising the importance of leaving a positive legacy when discussing climate change can effectively encourage policies and practices that reduce greenhouse gas emissions and promote behaviour change across all age groups [97,122,123]. The active involvement of older individuals in climate change activism can also yield community benefits, including a reduction in ageism, enhanced intergenerational connections, and the transfer of valuable knowledge. Furthermore, cultural wisdom and steadfast beliefs of older people have proven instrumental in enabling effective coping mechanisms during challenging situations, including disasters [124,125].

Investing in training for older adults in areas like political advocacy (such as letter writing, meeting with legislators, and personal storytelling) may hold significant potential for generating a multiplier effect within their local communities [126,127]. Climate change education, digital literacy programmes, and daily practices aimed at reducing carbon emissions could be integrated into older people's associations (OPAs) throughout East and Southeast Asia, following the model proposed by HelpAge International [97], and possibly expanded to other continents. Empowerment of older people will also lead to more advocacy at a governmental level to create adaptation plans that consider the needs of older people, and that hopefully will translate into increased research funding to address the existing evidence gaps.

Various policies aimed at mitigating climate change, such as those addressing fuel poverty e.g. inability to adequately meet household energy needs [128], renewable technologies, and insulation, prove advantageous for the well-being of older individuals [128,129]. The involvement of older adults in environmental volunteerism is a growing initiative. For instance, in Italy, an environmental volunteering programme involving park restoration showed increased life satisfaction, physical activity, and positive feelings among older people after they joined the activities [130].

The political situation of a given country influences the priority given to health, which is crucial for older people. The governmental allocation of funds to health – both addressing its determinants and to the health care system – determines how much health services can focus on the prevention and treatment of chronic illnesses among older people. National governments may not even recognise the need for the development of such strategies in low-income countries; however, international funding allocation and implementation of targeted programmes are timely needs.

### Research gaps

We have presented compelling evidence highlighting the substantial impact of climate change on older people worldwide. However, it is essential to recognise that much of this evidence is indirect, revealing gaps in our understanding of this issue. Notably, individuals who were actively engaged in the environmental movement, including the inaugural Earth Day in 1970, have now transitioned into the older population demographic. As global leaders age, there is an opportunity for them to leverage their leadership skills and social influence to foster intergenerational solidarity within the climate change movement, amplifying the voices of younger adults, as suggested by the UN Human Rights Council in 2021 [63].

Nevertheless, there is a noticeable lack of research exploring the opinions of older individuals regarding climate change and their motivations and contributions to climate change activism. Although many systematic reviews have been published on the impact of climate hazards on health, only a few focused specifically on older people, with evidence predominantly being systematically assessed for heat and air pollution. For example, Bunker and colleagues and Yu et al. assessed the impact of air temperature on older adults [131,132]. Most epidemiological studies on climate change impacts, with few exceptions, primarily target younger populations, and general adults, or even exclude older individuals entirely. This omission is concerning, given that older individuals likely bear a disproportionate burden of climate-related hazards and as the world population becomes increasingly older. Comprehensive evidence from large-scale longitudinal studies to imply causal inference and explore the cumulative effect of various hazard exposures is absent globally. The establishment of a universally accepted definition of climate hazards would facilitate cross-study comparisons.

Furthermore, studies involving older individuals often have a narrower scope, primarily focusing on morbidity and mortality rather than the enabling processes to optimise healthy ageing, including people’s functional ability and intrinsic capacity, which older individuals themselves often consider more critical than morbidities. It is imperative to integrate research on topics of interest to older individuals [92] and environmental sustainability to ensure the protection of the rights of older people, especially as the effects of the climate change crisis intensify. Additionally, research from low- and middle-income countries and vulnerable communities is lacking, such as from the Small Island States, with most of the evidence originating from North America, Australia, and Western Europe, even though the impact of climate change will disproportionately affect the Global South [133].

We have presented evidence highlighting the substantial impact of climate change on older people worldwide. However, it is essential to recognise that much of this evidence is indirect, revealing gaps in our understanding of this issue. One of the major limitations of this narrative review was the compressed timeframe for literature screening and report production. Although this study covers a broad scope encompassing multiple exposures, outcomes, and adaptation strategies relating to older adults, the short time frame affected our ability to present evidence systematically, including an assessment of its strength, risk of bias, and dose-response relationships. Furthermore, it is essential to acknowledge that although we tried to identify as many studies as possible, our systematic searches could have been further expanded by focusing on primary papers and not only systematic reviews when assessing the literature for each hazard identified.

We are hopeful for a shift towards greater inclusivity in climate change research. We advocate for prioritising research in the following areas:

•Studies that explicitly assess the impact of climate change on older people.

•Studies that focus on outcomes beyond mortality and morbidity, including functional ability and intrinsic capacity.

•Studies that assess impact across diverse regions of the world, including a focus on vulnerable groups within those areas.

•Studies that assess exposures and outcomes at multiple time points and the cumulative impacts of being exposed to different hazards.

•Studies that assess the inter-relationships between different hazards and quantify combined impacts on older people.

•Studies that focus on quantifying interactions and risks among groups with multiple vulnerabilities.

•Studies that value and quantify older people’s contributions to climate action that are not accounted for in standard economic indices, such as caregiving and volunteering, supporting people and sustaining the environment, as highlighted by the WHO Council on the Economics of Health for All [134].

•Studies that address ways to improve housing to enable older persons to age in place.

•Evaluations of how non-governmental climate action organisations empower and engage older people and promote intergenerational dialogue.

•Studies that identify effective ways to increase climate change literacy of older people.

There is a crucial need to harmonise the concerns of older individuals and environmental sustainability to safeguard the rights of older people, particularly given the escalating impacts of the climate change crisis [91]. Additionally, we must persist in developing climate change adaptation and mitigation solutions that are both equitable and culturally appropriate, with a specific focus on older people.

## CONCLUSIONS

If the increase in temperature is not kept within the levels decided in the Paris Agreement, the implications for our world will be devastating, with effects affecting all spheres of society, every living creature, and the ecosystem. Climate change is already having a large impact on older people’s lives, and with a projected increase in the number and proportion of older people worldwide, the scale of those impacts will increase even further.

The potential redirection of financial investments by local government and international organisations toward climate-friendly initiatives, along with the adjustment of food and energy consumption patterns that older people can endorse, represents a potent tool for positive impact investing in climate change initiatives for older individuals. For example, energy-efficient technologies provide dual advantages – cost savings to older people and positive environmental impact. [122,135]. Investment in training older people to be effective environmental activists can maximise their abilities to contribute to society and the value of their actions [56]. For example, in China, there are over half a million older people’s associations (OPAs) that let older people take the lead on environmental projects and climate education in both urban and rural communities [97]. Policymakers need to prioritise the most vulnerable, aligning local and global development and climate actions while disseminating public information on risks and solutions. Equitable adaptation strategies that reach all people are also crucial, ensuring that all individuals, regardless of socioeconomic status, have the resources and support to cope with climate-related challenges. For instance, targeted outreach and education programmes can bridge the gap, fostering resilience in communities facing diverse socioeconomic circumstances.

This review highlights specific entry points for policies and actions, emphasising potential leadership roles by either the health sector or other relevant domains globally. The objective is to systematically address determinants and diminish inequities, supporting individuals in achieving and maintaining optimal well-being as they age. Furthermore, the framework delineates essential features that health systems can integrate to amplify equity in the context of healthy ageing and alleviate the enduring repercussions of disparities. Essentially, the framework serves as a compass guide for formulating and executing effective strategies to advance healthy ageing and reduce health disparities.

Although older people are overwhelmingly affected by climate change, existing evidence on this group is limited, and more is needed to understand the multifaceted impacts and the complex network of exposure pathways underlying these effects. What is clear is that older people are not a homogenous group and that specific individual, and contextual characteristics put certain people at a heightened risk. More research, however, is needed to understand the relationship between different vulnerabilities and the implications of having multiple vulnerabilities.

Older people play a crucial role in driving these changes, and there is a growing momentum to increase climate change activism among this demographic, as well as their awareness and understanding of climate change issues. By actively involving older individuals in conversations about climate change and creating age-friendly environments, we not only have the opportunity to mitigate the adverse effects of climate change on healthy ageing but also to transform population ageing into a valuable resource for addressing the most pressing challenge of our time.

## Additional material


Online Supplementary Document
