# Supplementary material for: Climate change and healthy ageing: An assessment of the impact of climate hazards on older people
Source: J Glob Health. 2024 May 24;14:04101. doi: 10.7189/jogh.14.04101 (PMC11116931; doi:10.7189/jogh.14.04101)

Online Supplementary Material

Figure S1: Framework for the impact of extreme temperatures on older people

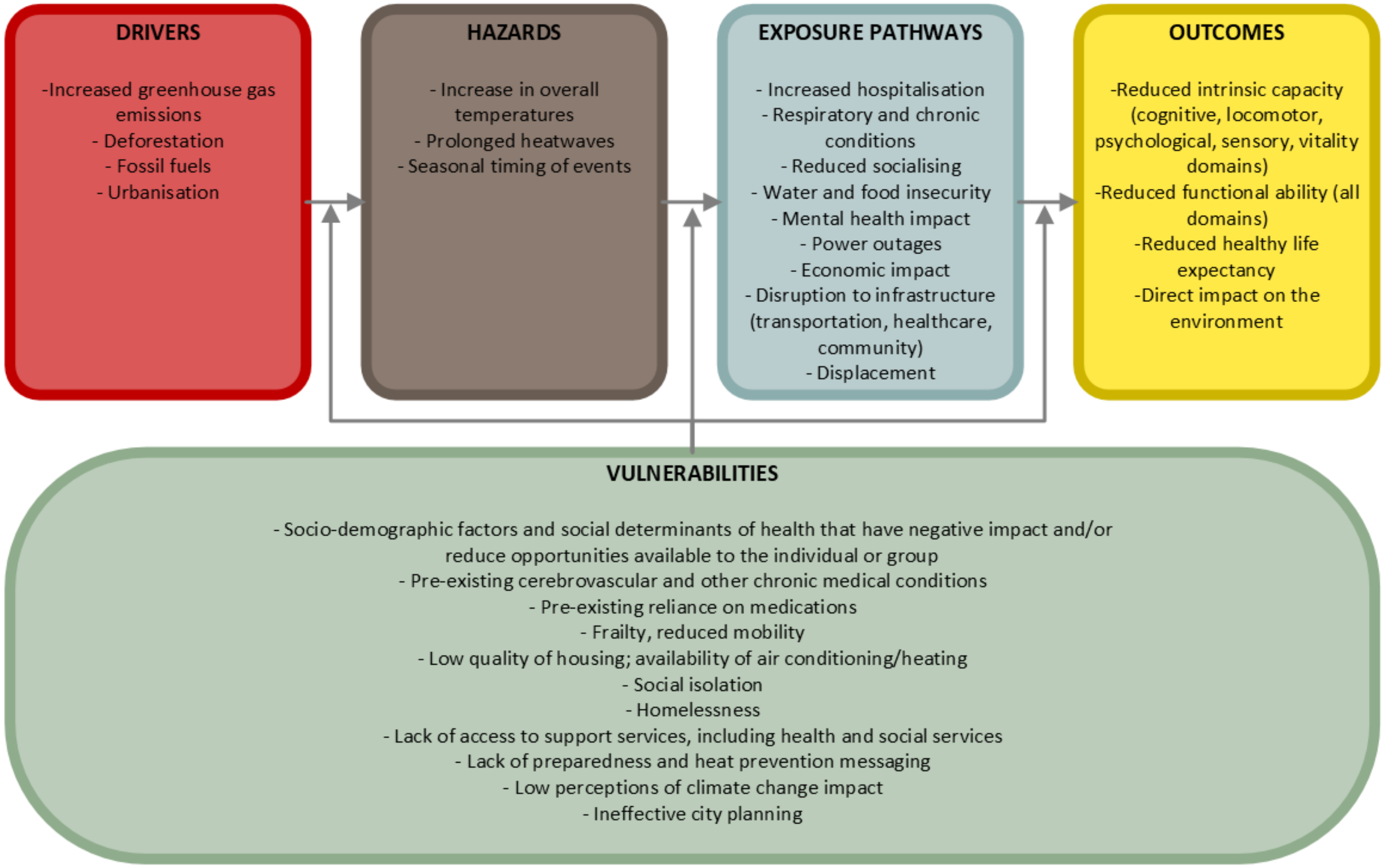

Figure S2: Framework for the impact of wildfires on older people

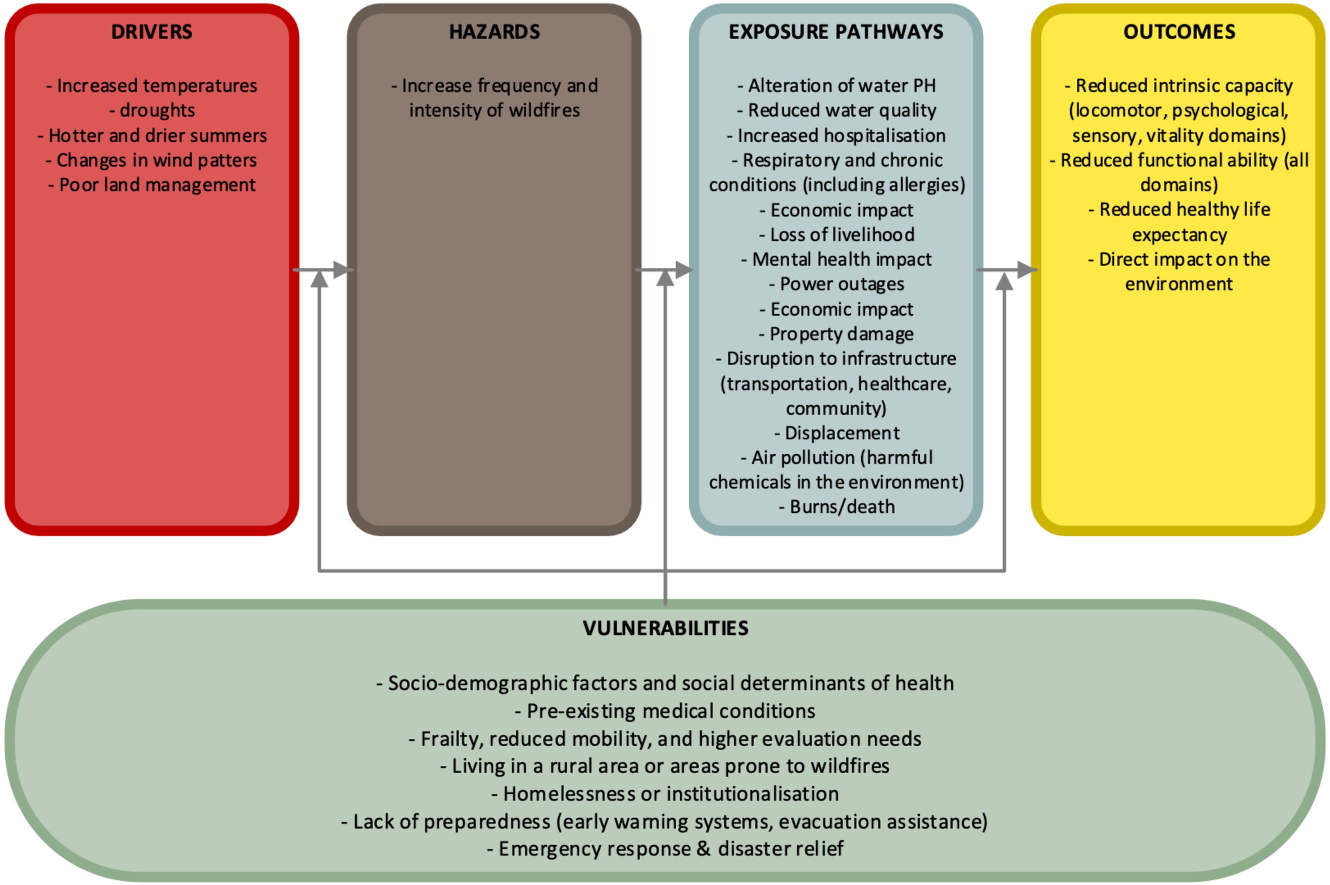

Figure S3: Framework for the impact of droughts on older people

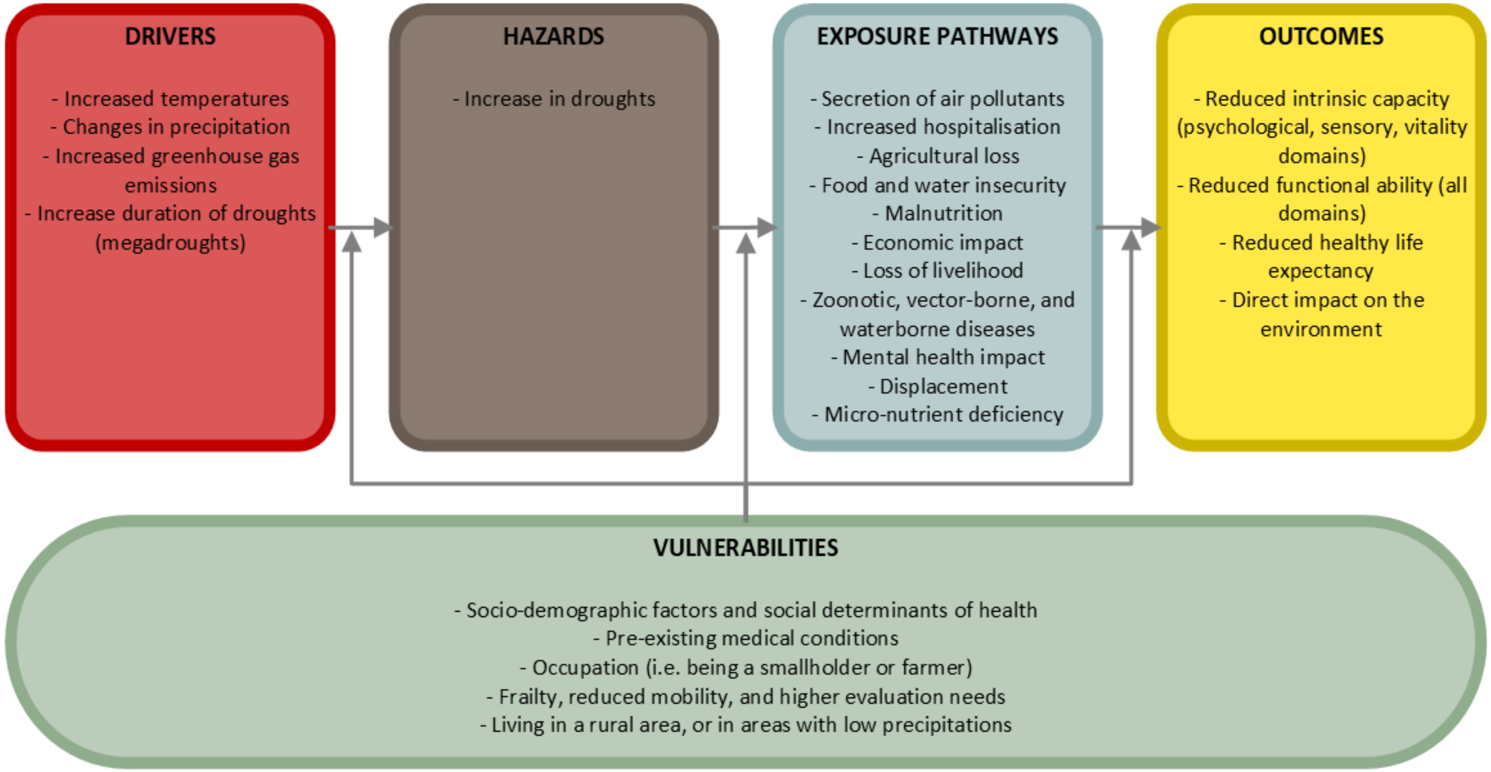

Figure S4: Framework for the impact of flooding, storms, and sea-level rise on older people

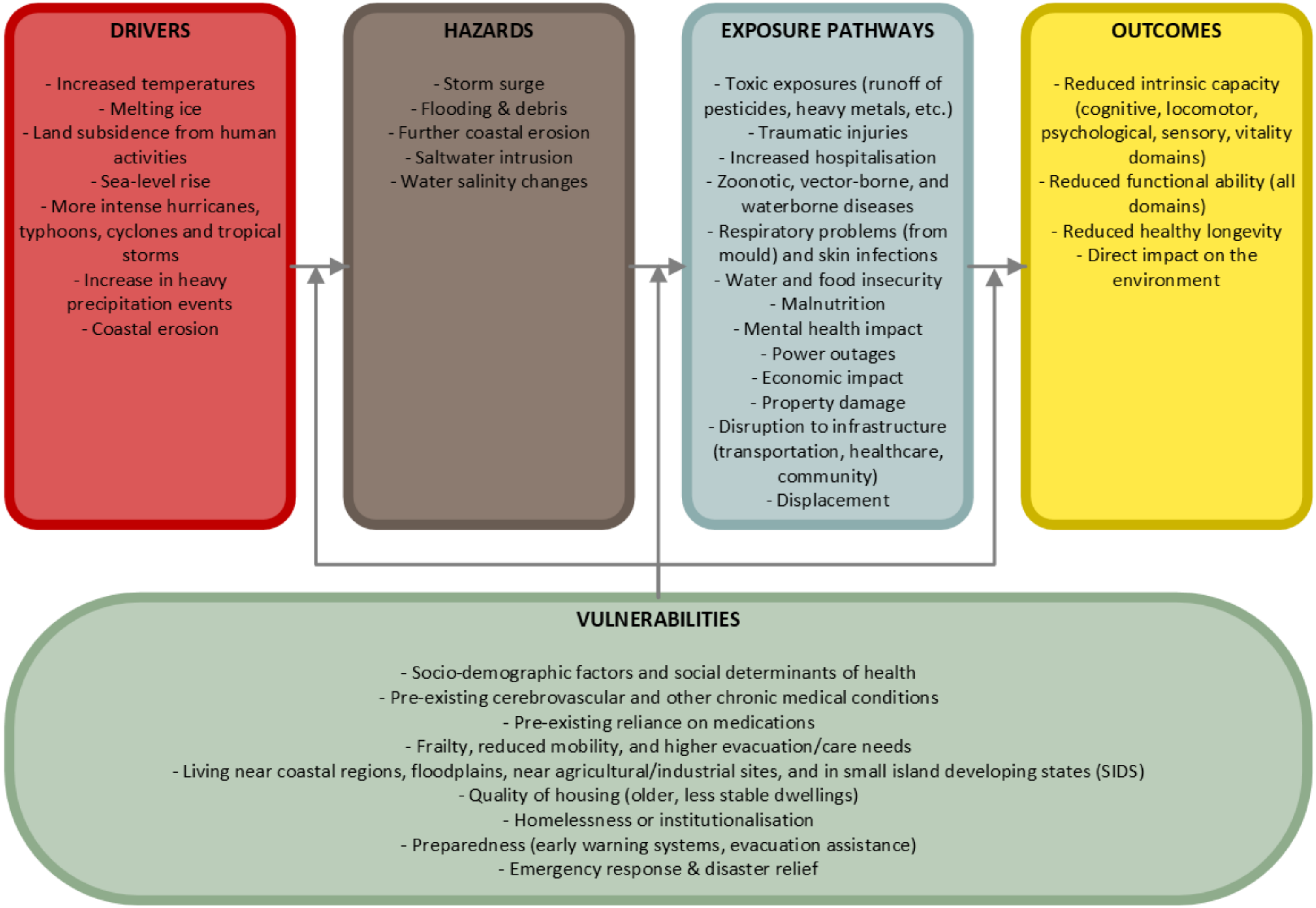

Figure S5: Framework for the impact of poor air quality on older people

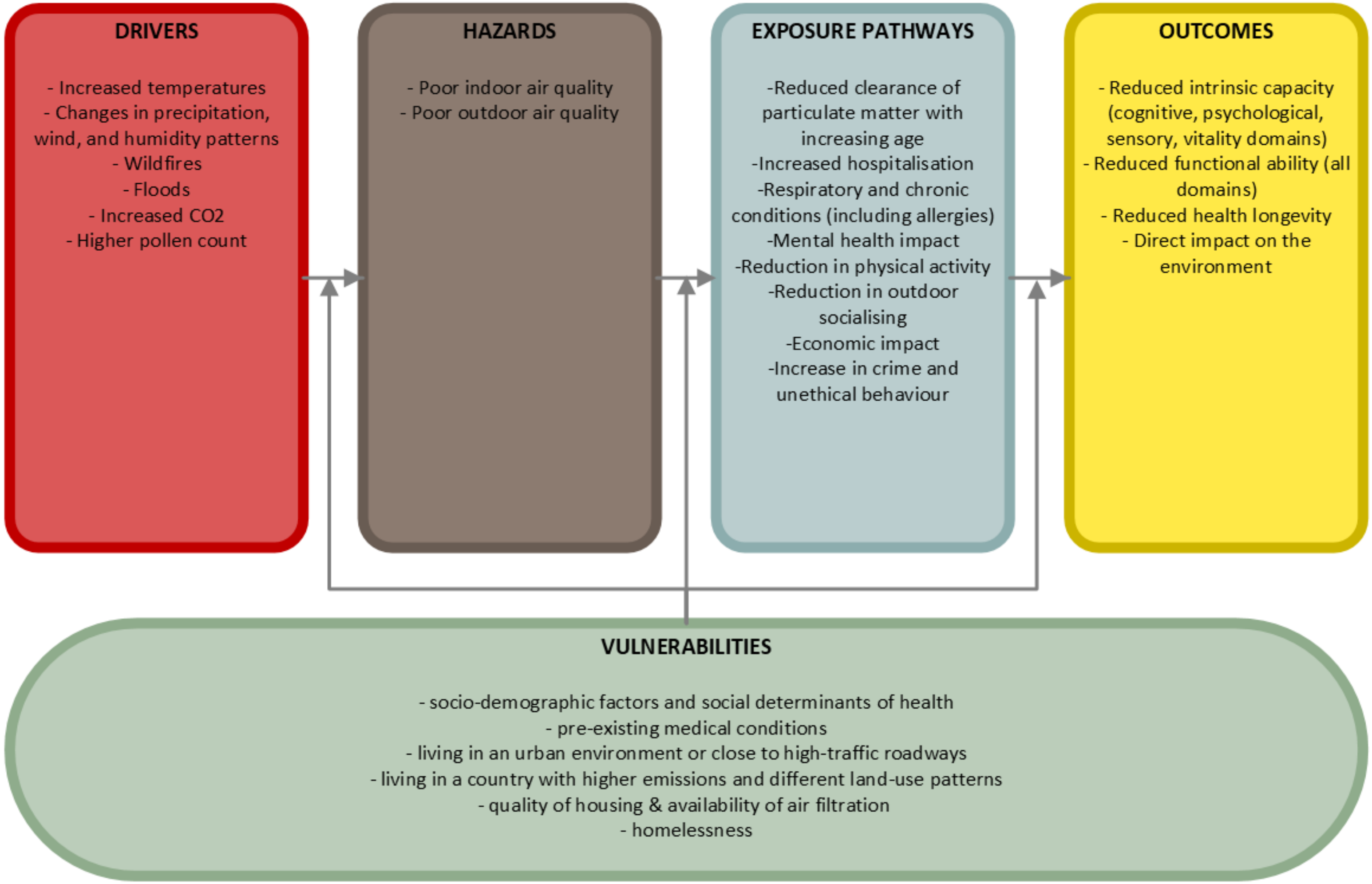

Figure S6: Framework for the impact of climate-sensitive infectious diseases on older people

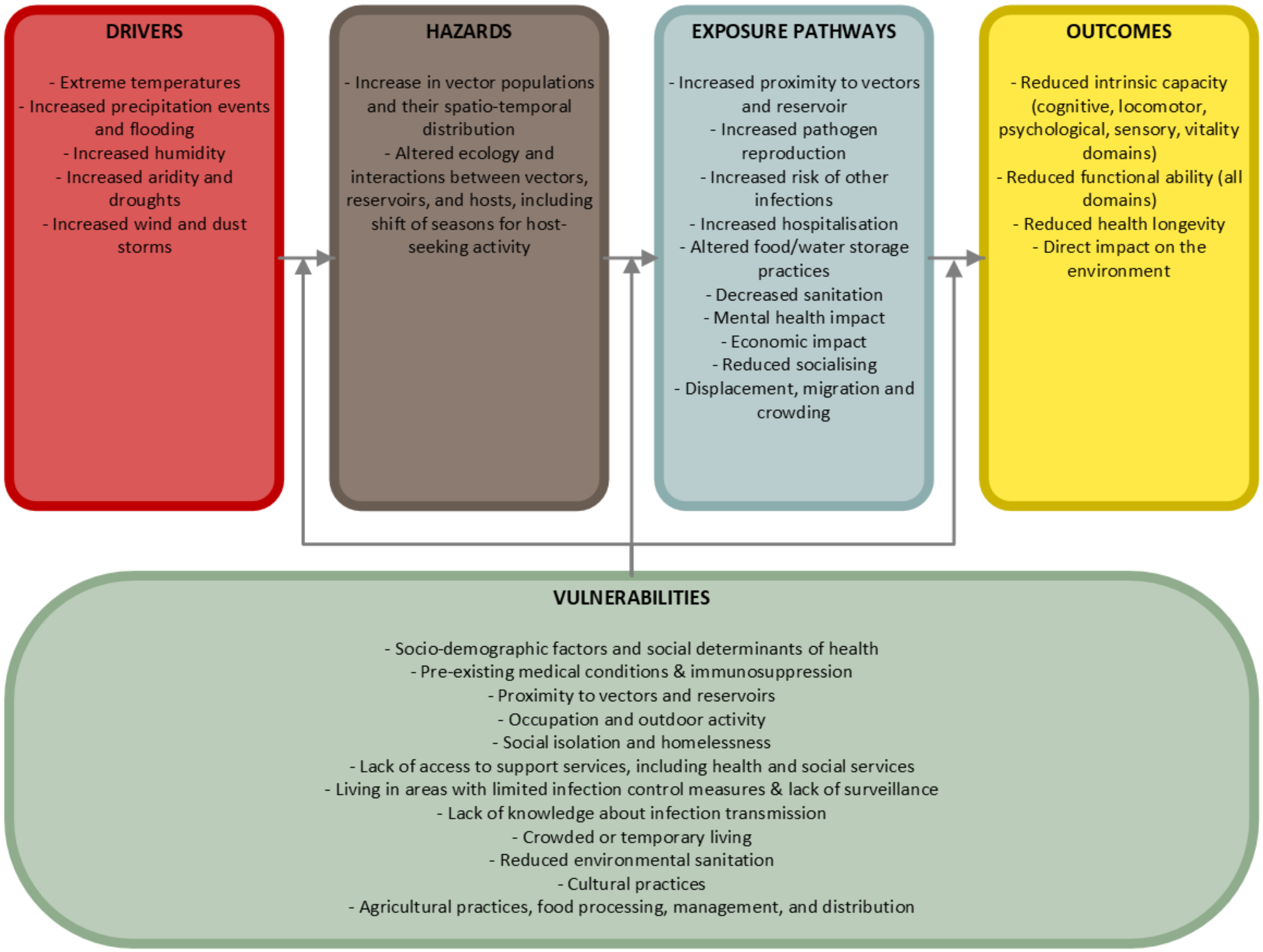

Supplement: Online Supplementary Document [file jogh-14-04101-s001.pdf]
